# Biocatalyzed Synthesis of Benzoyl and Cinnamoylamides Inspired by Rice Phytoalexins

**DOI:** 10.1021/acsagscitech.4c00380

**Published:** 2025-02-24

**Authors:** Cecilia Pinna, Luca Nespoli, Giulia Brioschi, Andrea Kunova, Paolo Cortesi, Piera Anna Martino, Francesco Molinari, Loana Musso, Sabrina Dallavalle, Martina L. Contente, Andrea Pinto

**Affiliations:** † Department of Food, Environmental and Nutritional Sciences (DeFENS), University of Milan, via Celoria 2, 20133 Milan, Italy; ‡ Department of Biomedical, Surgical and Dental Sciences (DSBCO), One Health Unit, University of Milan, via Pascal 36, 20133 Milan, Italy

**Keywords:** rice phytoalexins, benzoylamides, cinnamoylamides, antimicrobial agents, CaL-B, biocatalyzed reactions, green chemistry

## Abstract

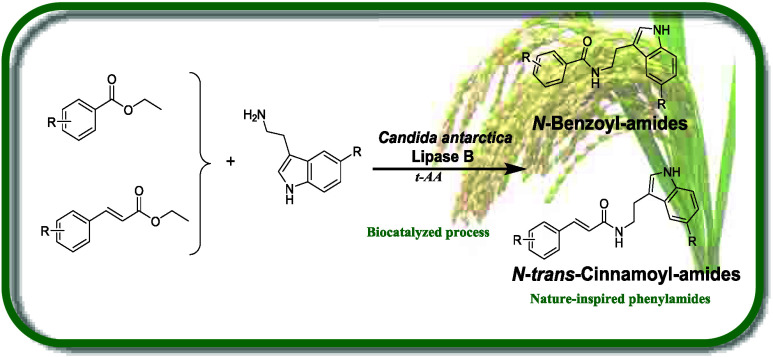

Worldwide, phytopathogenic fungi, bacteria, and viruses are responsible for huge crop losses each year, threatening agricultural progress and food security and causing massive economic damages. *Pyricularia oryzae* represents one of the most dangerous fungal phytopathogens being the cause of rice blast, a highly destructive disease widely distributed across the world. In this critical context, good agricultural practices necessarily need to be supported using novel, effective, and sustainable agrochemicals. It is known that plants naturally counteract exogenous infections by synthesizing defense secondary metabolites, known as phytoalexins. Inspired by *N*-benzoyltryptamine and *N*-cinnamoyltryptamine, two phytoalexins found in *Oryza sativa*, we designed a collection of tryptamine-based derivatives. The compounds were synthesized exploiting an enzymatic approach, using *Candida antarctica Lipase B* (CaL-B) as a biocatalyst and *tert*-amyl alcohol (*t*-AA) as an unconventional green solvent. The activity was evaluated against a panel of different phytopathogenic fungi as well as selected Gram-negative and Gram-positive bacteria. The obtained results pave the way for novel nature-inspired products as a valuable alternative to currently available pesticides.

## Introduction

**Figure 1:**
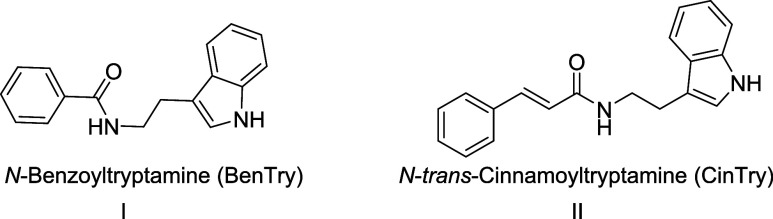
General structure of the two main classes of rice phenylamides.

Rice is a staple food for more than half of the world population, particularly in East and South Asia, Latin America, and some areas of the African continent.^1^ In Europe, rice is mainly produced in the Mediterranean countries, with Italy being the major producer.^2^ Nowadays, rice growing sector is facing several challenges such as global population growth and ecological factors (*e.g.*, climate change, flooding, droughts, storms) responsible for significant damage and losses of agricultural crops. Nevertheless, the main threat to agricultural progress, and food security as well, is represented by plant diseases, arising from biotic or abiotic stress and responsible for the impairment of plant growth and/or development and, eventually, for significant harvest losses. Phytopathogenic fungi, bacteria and viruses represent the major causes of plant biotic stress. It is estimated that crop losses caused worldwide by these phytopathogens range between 20 and 40%,^3^ with *Pyricularia oryzae* at the top of the most dangerous phytopathogenic fungi affecting rice and other cereal crops.^4,5^*P. oryzae* is responsible for rice blast, a highly destructive disease widespread in Europe, Asia, Central and South America, Sub-Saharan Africa and Australia. Rice blast can cause yield losses of up to 30% (even complete loss in favorable conditions of temperature and humidity).^6,7^ According to a survey carried out in 2010, yearly rice losses due to blast disease alone would be sufficient to feed 60 million people.^8^ In addition, an ever decreasing number of fungicides are available to date for its management. For instance, with the recent withdrawal of tricyclazole from European Union, only four fungicide classes remain available for rice disease management, particularly for the treatment of *P. oryzae* infections: strobilurins (quinone outside inhibitors, QoI) and demethylation inhibitors (DMI) as synthetic fungicides, whereas sulfur and *Bacillus subtilis* QST 713 also in biological agriculture.^9^ Such a limited choice of fungicides leads to their repeated use, thus increasing the risk of resistance phenomena. This is particularly true for fungicides with a single-site mode of action such as strobilurins, which in the latest years became less effective due to mutations in the molecular target (the cyt *bc1* complex of the mitochondrial respiratory chain).^10^ In this critical context, good agricultural practices necessarily need to be supported by the use of novel effective agrochemicals. It is known that plants naturally counteract exogenous infections by synthesizing a large number of structurally diverse endogenous antimicrobials, known as phytoalexins.^11,12^ Natural phytoalexins are not produced by plant basal metabolism but are specifically biosynthesized in small quantities in response to external pathogen infections.^11^ In a recent review, Valletta and co-workers exhaustively described the different classes of rice phytoalexins: (i) diterpenes, such as momilactones, phytocassanes and oryzalexins; (ii) flavonoids as sakuranetin and (iii) phenylamides.^13^ The latter group is composed of amidic compounds resulting from the conjugation of acids (*e.g.*, benzoic, cinnamic, caffeic, ferulic and *p*-coumaric acid) with arylmonoamines such as tyramine, tryptamine and serotonin.^14^ Two main classes can be identified: benzoylamides (**I**) and cinnamoylamides (**II**) (e.g., general structures shown in Figure 1).

The plant production of cinnamoylamides begins when a pathogen infection occurs. It exploits the phenylpropanoid pathway starting from phenylalanine deamination catalyzed by phenylalanine ammonia lyases (PALs) to obtain *trans*-cinnamic acid, followed by its esterification with CoA. The final condensation with endogenous amines is catalyzed by different cinnamoyl transferases (CTs), acting on biogenic amines (originated from amino acid metabolism) and various cinnamoyl CoA-thioesters.^15^ A recent study demonstrated that genes encoding for some particular CTs - namely, agmatine hydroxycinnamoyl transferases (OsAHT1/2) and tryptamine hydroxycinnamoyl transferases (OsTBT1/2) - are induced in rice plants during *P. oryzae* infection.^16^ In fact, several phenylamides were found in rice leaves infected by the blast fungus *P. oryzae* and the rice brown spot fungus *Bipolaris oryzae*, including *N*-*p*-coumaroylserotonin (CouSer), *N*-feruloyltryptamine (FerTry), and *N*-feruloylserotonin (FerSer).^17,18^

Indeed, the overexpression of cinnamoyl transferases enhances the rice resistance against phytopathogens modulating the production of phenylamides.^19^ Prompted by this evidence and by the scarce availability of phenylamides in natural matrices, we decided to prepare a small collection of nature-derived benzoyl and cinnamoylamides to investigate their potential application in the agri-food sector. Inspired by the biosynthetic pathway in rice plant, our efforts were devoted to setting up a sustainable biocatalytic strategy. To this purpose we selected the commercially available immobilized lipase from *Candida antarctica* (CaL-B) due to its ability to perform ammidation reactions in organic media well tolerating high temperatures.^20−22^ All the obtained compounds were tested for their inhibitory activity against wild-type and strobilurin-resistant *P. oryzae* strains. The biological evaluation was then extended to a broader panel of phytopathogenic fungi, including *Penicillium expansum*, *Aspergillus niger*, *Mucor pyriformis*, *Botrytis cinerea*, *Fusarium culmorum*, and *Fusarium graminearum*, as well as to a small collection of representative foodborne Gram-positive and Gram-negative bacteria, namely *Escherichia coli*, *Salmonella enterica* Enteritidis, *Pseudomonas aeruginosa*, and *Staphylococcus aureus*.

## Materials and Methods

### Materials

All reagents have been purchased from commercial suppliers and used without any further purification. ^1^H NMR and ^13^C NMR spectra were recorded with a Bruker AV600 (^1^H 600 MHz; ^13^C 150 MHz) or a Bruker AvanceTM NEO 400 MHz (^1^H 400 MHz; ^13^C 100 MHz) spectrometers. TMS was used as an internal standard, chemical shifts (δ) are expressed in ppm and coupling constants (*J*) in Hz. Chloroform-*d*, methanol-*d*_4_ and DMSO-*d*_6_ were used as deuterated solvents for NMR analysis. TLC analyses were performed using commercial silica gel 60 F_254_ aluminum sheets. Reference spots were revealed using a UV lamp (λ = 254 or 365 nm) or using the appropriate development reagents such as ninhydrin (1.5 g of ninhydrin, 3.0 mL of acetic acid in 100 mL of ethanol) or KMnO_4_ (1.5 g of KMnO_4_, 10 g K_2_CO_3_, and 1.25 mL 10% NaOH in 200 mL water). Isolation and purification of the compounds were performed by flash column chromatography on silica gel 60 (230–400 mesh). *Candida antarctica* (CaL-B) immobilized on acrylic resin (≥5000 U/g) and Tyrosinase from *Agaricus bisporus* (≥4 U/mg) were purchased from Merk Life Science S. r. l. (Milan, Italy).

### General Procedure for the Biocatalyzed Preparation of Benzoylamides **2a–c** and Cinnamoylamides **4a–c** and **5a–c**

**Figure 2:**
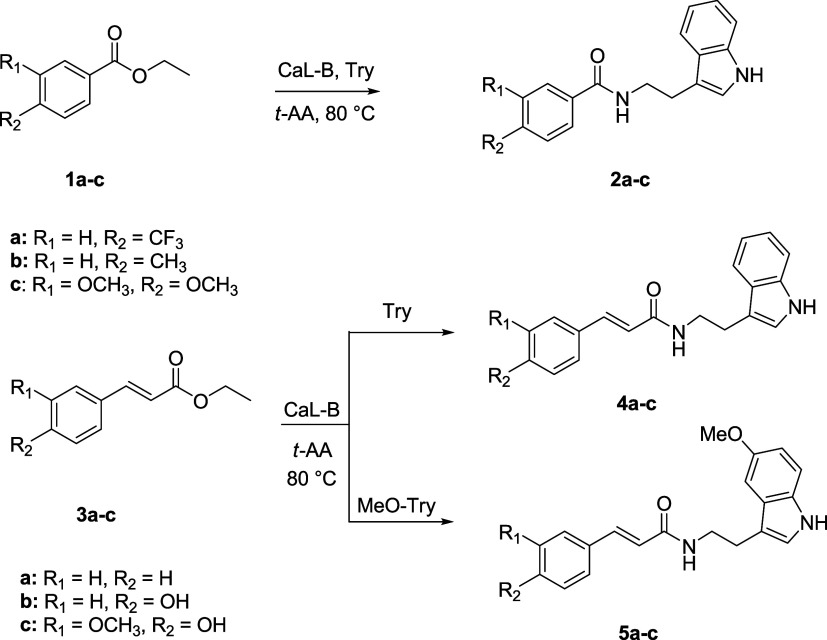
Biocatalyzed preparation of benzoylamides 2a–c and cinnamoylamides 4a–c/5a–c.

In 10 mL screw-cap tubes, a solution of 0.25 M (0.5 mmol) of the proper ethyl ester in *tert*-amyl alcohol (*t*-AA) was prepared. The selected amine (0.5 M, 1 mmol) was then added together with CaL-B (50 mg/mL, 250 U/mL) and molecular sieves. The obtained reaction mixture (final volume: 2 mL) was kept under gentle shaking at 80 °C for 24 h. Then, CaL-B was removed by filtration. The solvent was evaporated under reduced pressure. The crude products were purified by flash chromatography as described below (Figure 2).

#### *N*-(2-(1H-Indol-2-yl)ethyl)-4-(trifluoromethyl)benzamide **(2a)**

Purification by flash chromatography (cyclohexane/ethyl acetate 7:3) gave the desired product as a white powder in 77% yield. R_f_ (CHX/EtOAc 7:3) = 0.48. ^1^H NMR (600 MHz, CDCl_3_) δ: 8.09 (bs, 1H), 7.75 (d, *J* = 8.0 Hz, 2H), 7.64 (d, *J* = 8.0 Hz, 2H), 7.64 (d, *J* = 7.5 Hz, 1H), 7.40 (d, *J* = 7.5 Hz, 1H), 7.23 (t, *J* = 7.5 Hz, 1H), 7.14 (t, *J* = 7.5 Hz, 1H), 7.08 (s, 1H), 6.22 (bs, 1H), 3.82 (dt, *J* = 6.5, 6.5 Hz, 2H), 3.12 (t, *J* = 6.5 Hz, 2H). ^13^C NMR (150 MHz, CD_3_OD): δ 168.8, 139.6, 138.2, 133.9 (q, *J* = 33 Hz), 129.0, 128.8 (×2C), 126,4 (×2C), 125.30 (q, *J* = 275 Hz), 123.5, 122.4, 119.6, 119.3, 113.3, 112.3, 42.3, 26.2.

#### *N*-(2-(1*H*-Indol-3-yl)ethyl)-4-methylbenzamide **(2b**)

Purification by flash chromatography (cyclohexane/ethyl acetate, 7:3) gave the desired product as a white solid in 30% yield. R_f_ (DCM/MeOH 98/2 + 0.2% TEA) = 0.48. ^1^H NMR (600 MHz, CDCl_3_) δ: 8.31 (s, 1H), 7.65 (d, *J* = 8.0 Hz, 1H), 7.58 (d, *J* = 8.0 Hz, 2H), 7.38 (d, *J* = 8.0 Hz, 1H), 7.22 (t, *J* = 8.0, 1H), 7.17 (d, *J* = 8.0 Hz, 2H), 7.13 (t, *J* = 8.0 Hz, 1H), 7.04 (s,1H), 6.26 (bs, 1H), 3.80 (dt, *J* = 6.3, 6.3 Hz, 2H), 3.09 (t, *J* = 6.6 Hz, 2H), 2.37 (s, 3H). ^13^C NMR (150 MHz, CDCl_3_): δ 167.6, 141.9, 136.6, 131.9, 129.3 (×2C), 127.5, 127.0 (×2C), 122.3, 122.3, 119.6, 118.9, 113.1, 111.5, 40.4, 25.5, 21.5.

#### *N*-(2-(1*H*-Indol-3-yl)ethyl)-3,4-dimethoxybenzamide **(2c)**

Purification by flash chromatography (cyclohexane/ethyl acetate, 7:3) gave the desired product as a white solid in 15% yield. R_f_ (DCM/MeOH 95/5+ 0.2% TEA) = 0.32. ^1^H NMR (600 MHz, CDCl_3_) δ: 8.18 (bs, 1H), 7.65 (d, *J* = 8.0 Hz, 1H), 7.38 (d, *J* = 8.0 Hz, 1H), 7.34 (s, 1H), 7.23 (t, *J* = 8.0 Hz, 1H), 7.13 (t, *J* = 8.0 Hz, 1H), 7.13 (d, *J* = 8.0 Hz, 1H), 7.06 (s, 1H), 6.78 (d, *J* = 8.0 Hz, 1H), 6.20 (bs, 1H), 3.89 (s, 3H), 3.86 (s, 3H), 3.79 (dt, *J* = 6.2, 6.2 Hz, 2H), 3.09 (t, *J* = 6.2 Hz, 2H). ^13^C NMR (150 MHz, CDCl_3_): δ 167.0, 151.6, 148.9, 136.4, 127.4, 127.4, 122.3, 122.1, 119.6, 119.2, 118.8, 113.2, 111.3, 110.5, 110.2, 56.0, 55.9, 40.4, 25.3.

#### *N*-(2-(1*H*-Indol-3-yl)ethyl)cinnamamide **(4a)**

Purification by flash chromatography (gradient cyclohexane/ethyl acetate 8:2 to 45:55) gave the desired product as a beige powder in 50% yield. R_f_ (DCM/MeOH 100/2) = 0.71. ^1^H NMR (600 MHz CDCl_3_) δ 8.24 (bs, 1H), 7.64 (d, *J* = 8.0 Hz, 1H), 7.62 (d, *J* = 15.6 Hz, 1H), 7.49–7.43 (m, 2H), 7.39 (d, *J* = 8.0 Hz, 1H), 7.36–7.31 (m, 3H), 7.22 (t, *J* = 8.0 Hz, 1H), 7.14 (t, *J* = 8.0 Hz, 1H), 7.07 (s, 1H), 6.30 (d, *J* = 15.6 Hz, 1H), 5.71 (bs, 1H), 3.75 (dt, *J* = 5.9, 6.8 Hz, 2H), 3.0 (t, *J* = 6.8 Hz, 2H). ^13^C NMR (150 MHz, CDCl_3_): δ 166.0, 141.1, 136.6, 135.0, 129.8, 128.9 (×2C), 127.9 (×2C), 127.5, 122.4, 122.3, 120.9, 119.7, 118.9, 113.2, 111.4, 40.1, 25.5.

#### (E)-*N*-(2-(1*H*-Indol-3-yl)ethyl)-3-(4-hydroxyphenyl)acrylamide **(4b)**

Purification by flash chromatography (gradient cyclohexane/ethyl acetate 7:1 to 1:1) afforded the desired product as a brown powder in 28% yield. *R*_f_ (DCM/MeOH 95/5) = 0.26. ^1^H NMR (600 MHz CDCl_3_) δ 8.32 (bs, 1H), 7.65 (d, *J* = 8.0 Hz, 1H), 7.62 (d, *J* = 15.5 Hz, 1H), 7.47–7.43 (m, 2H), 7.39 (d, *J* = 8.0 Hz, 1H), 7.37–7.31 (m, 3H), 7.22 (t, *J* = 8.0 Hz, 1H), 7.11 (t, *J* = 8.0 Hz, 1H), 7.03 (s, 1H), 6.23 (d, *J* = 15.5 Hz, 1H), 5.84 (bs, 1H), 3.74 (dt, *J* = 6.7, 6.7 Hz, 2H), 3.05 (t, *J* = 6.7 Hz, 2H). ^13^C NMR (150 MHz, CDCl_3_): δ 166.2, 141.0, 136.5, 134.8, 129.7, 129.7 (×2C), 128.8 (×2C), 127.8, 122.3, 122.2, 120.8, 119.5, 118.7, 112.7, 111.4, 40.1, 25.3.

#### (E)-*N*-(2-(1*H*-Indol-3-yl)ethyl)-3-(4-hydroxy-3-methoxyphenyl)acrylamide **(4c)**

Purification by column chromatography (dichloromethane/methanol 110:2) afforded the desired product as a white powder in 21% yield. *R*_f_ (CHX/EtOAc 1/1) = 0.29 ^1^H NMR (600 MHz CD_3_OD) δ 10.23 (bs, 1H), 8.08 (bs, 1H), 7.61 (d, *J* = 7.9 Hz, 1H), 7.47 (d, *J* = 15.6 Hz, 1H), 7.36 (d, *J* = 7.9 Hz, 1H), 7.19–7.08 (3H, m), 7.07–6.99 (2H, m), 6.83 (d, *J* = 8.4 Hz), 6.44 (d, *J* = 15.6 Hz, 1H), 3.90 (s, 3H), 3.69–3.56 (m, 2H), 3.04 (t, *J* = 6.3 Hz, 2H). ^13^C NMR (150 MHz, CD_3_OD): δ 169.2, 149.8, 149.3, 141.9, 138.2, 128.8, 128.3, 123.4, 123.2, 122.3, 119.6, 119.3, 118.9, 116.4, 113.3, 112,2, 111.6, 56.4, 41.6, 26.4.

#### *N*-(2-(5-Methoxy-1*H*-indol-3-yl)ethyl)cinnamamide **(5a)**

Purification by flash chromatography (gradient cyclohexane/ethyl acetate 7:3 to 1:1) afforded the desired product as a yellow powder in 83% yield. *R*_f_ (DCM/MeOH 98/2) = 0.42. ^1^H NMR (600 MHz CDCl_3_) δ 8.42 (bs, 1H), 7.65 (d, *J* = 15.5 Hz, 1H), 7.44–7.38 (m, 2H), 7.34–7.29 (m, 3H), 7.27 (d, *J* = 8.7 Hz, 1H), 7.08 (s, 1H), 7.02 (d, *J* = 1.8 Hz, 1H), 6.90 (dd, *J* = 1.8, 8.7 Hz, 1H), 6.35 (d, *J* = 15.5 Hz, 1H), 6.00 (bs, 1H), 3.84 (s, 3H), 3.74 (dt, *J* = 6.6, 6.6 Hz, 2H), 3.03 (t, *J* = 6.6 Hz, 2H).^13^C NMR (150 MHz, CD_3_OD): δ 166.1, 154.2, 141.0, 134.9, 131.7, 129.7, 128.9 (×2C), 127.9 (×2C), 127.9, 123.1, 120.9, 112.8, 112.6, 112.2, 10064, 56.0, 40.2, 25.5.

#### (E)-3-(4-Hydroxyphenyl)-*N*-(2-(5-methoxy-1*H*-indol-3-yl)ethyl)acrylamide **(5b)**

Purification by column chromatography (dichloromethane/methanol 98:2) afforded the desired product as a white powder in 30% yield. R_f_ (DCM/MeOH 98/2) = 0.55.^1^H NMR (600 MHz CH_3_OH-*d*_4_) δ 7.45 (d, *J* = 16.0 Hz, 1H), 7.41–7.32 (m, 2H), 7.21 (d, *J* = 8.7 Hz, 1H), 7.09–7.03 (m, 2H), 6.81–6.75 (2H, m) 6.87 (dd, *J* = 8.8, 2.2 Hz, 1H), 6.73 (dd, 1H, *J* = 2.3, 8.7 Hz), 6.38 (d, *J* = 16.0 Hz, 1H), 3.80 (s, 3H), 3.58 (t, *J* = 7.3 Hz, 2H), 2.97 (t, *J* = 7.3 Hz, 2H).

^13^C NMR (150 MHz, CDCl_3_): δ 166.1, 154.1, 141.0, 134.9, 131.7, 129.7, 128.9 (×2C), 127.9 (×2C), 127.9, 123.1, 121.0, 112.7, 112.5, 112.2, 100.6, 56.0, 40.2, 25.4.

#### (E)-3-(4-Hydroxy-3-methoxyphenyl)-*N*-(2-(5-methoxy-1*H*-indol-3-yl)ethyl)acrylamide **(5c)**

Purification by column chromatography (dichloromethane/methanol 98:2) afforded the desired product as a white powder in 15% yield. *R*_f_ (DCM/MeOH 95/5) = 0.55. ^1^H NMR (600 MHz CDCl_3_) δ 7.95 (s, 1H), 7.92 (d, *J* = 15.0 Hz, 1H), 7.28 (d, *J* = 8.8 Hz, 1H), 7.08–7.04 (m, 2H), 7.02 (dd, *J* = 8.0. 1.2 Hz, 1H), 6.95 (s, 1H), 6.90–6.85 (m, 2H), 6.15 (d, *J* = 15.0 Hz, 1H), 5.78 (bs, 1H), 5.63 (s, 1H), 3.90 (s, 3H), 3.84 (s, 3H), 3.76–3.71 (dt, *J* = 6.5, 6.5 Hz, 2H), 3.49 (s, 3H), 3.02 (t, *J* = 6.5 Hz, 2H).^13^C NMR (150 MHz, CDCl_3_): δ 166.3, 154.3, 147.5, 146.8, 141.0, 131.7, 128.0, 127.5, 123.0, 122.3, 118.5, 114.8, 113.1, 112.8, 112.1, 109.7, 100.6, 56.1 (×2C), 40.2, 25.6.

### General Procedure for the Biocatalyzed Preparation of Cinnamoylamides **4d** and **5d**

The proper cinnamoylamide (**4b** or **5b**, 0.05 mmol) was suspended in a mixture of 0.1 M phosphate buffer (pH 8.0) and DMSO (10% v/v). Ascorbic acid (3 eq, 0.15 mmol) was added, followed by a solution of tyrosinase in phosphate buffer (1 mg/mL, 233 μL, 4 U/mL). The obtained suspension was stirred at room temperature for 3 days. After this time, the reaction mixture was extracted three times with ethyl acetate. The organic layers were dried over Na_2_SO_4_, filtered, and the solvent was evaporated under reduced pressure. Thre crude products were purified by flash chromatography affording the desired product as described below.

#### (E)-*N*-(2-(1*H*-Indol-3-yl)ethyl)-3-(3,4-dihydroxyphenyl)acrylamide (**4d**)

Purification by flash chromatography (gradient cyclohexane/ethyl acetate 1:1 to 15:85) afforded the desired product as an orange powder in 10% yield. R_f_ (DCM/MeOH 95/5) = 0.21. ^1^H NMR (600 MHz CD_3_OD) δ 7.60 (d, *J* = 8.0 Hz, 1H), 7.42 (d, *J* = 15.6 Hz, 1H), 7.35 (d, *J* = 8.0 Hz, 1H), 7.08–7.03 (m, 2H), 7.01–6.95 (m, 2H), 6.80 (dd, *J* = 8.3, 1.2 Hz, 1H), 6.78 (d, *J* = 8.3 Hz, 1H), 6.37 (d, *J* = 15.6 Hz, 1H), 3.61 (t, *J* = 7.3 Hz, 2H), 3.02 (t, *J* = 7.3 Hz, 2H). ^13^C NMR (150 MHz, CD_3_OD): δ 169.3, 148.7, 146.7, 142.1, 138.2, 128.8, 128.4, 123.4, 122.3, 122.1, 119.6, 119.3, 118.6, 116.4, 115.0, 113.3, 112.2, 41.6, 26.4.

#### (E)-3-(3,4-Dihydroxyphenyl)-*N*-(2-(5-methoxy-1*H*-indol-3-yl)ethyl)acrylamide (**5d**)

Purification by flash chromatography (gradient cyclohexane/ethyl acetate 7:3 to 3:7) afforded the desired product as a brown powder in 15% yield. R_f_ (DCM/MeOH 95/5) = 0.20. ^1^H NMR (600 MHz CD_3_OD) δ 7.40 (d, *J* = 15.7 Hz, 1H), 7.23 (d, *J* = 8.7 Hz, 1H), 7.09–7.01 (m, 2H), 7.02 (s, 1H), 6.91 (d, *J* = 7.8 Hz, 1H), 6.79–6.68 (m, 2H), 6.38 (d, *J* = 15.7 Hz, 1H), 3.82 (s, 3H), 3.61 (t, *J* = 6.8 Hz, 2H), 2.99 (t, *J* = 6.8 Hz, 2H).^13^C NMR (150 MHz, CD_3_OD): δ 169.3, 154.9, 148.7, 146.7, 142.1, 133.4, 129.3, 128.3, 124.2, 122.1, 118.6, 116.5, 115.1, 113.2, 112.9, 112.7, 101.3, 56.4, 41.7, 26.4.

### Bacterial Strains and Culture Conditions

*E. coli* ATCC 25922 (*Ec*), *S. enterica* Enteritidis ISM 8324 (*Se*), *P. aeruginosa* IMV 1 (*Pa*) and *Staphylococcus aureus* ATCC 6538 (*Sa*), after thawing and a first inoculation in Tryptic Soy broth (TSB, Thermo Fisher Diagnostics S.p.A, Rodano, Italy) for 24 h at 37 °C, were plated on Tryptic Soy Agar +5% sheep blood (Microbiol, Italy) and incubated aerobically at 37 °C for 24 h.

### Determination of the Minimum Inhibitory Concentration

The minimum inhibitory concentration (MIC) was determined using the microdilution assay, according to the Clinical and Laboratory Standards Institute (CLSI) guidelines.^23^ All strains were grown on Tryptic Soy Agar (Thermo Fisher Diagnostics S.p.A, Rodano, Italy) and 3 or 4 isolated colonies were suspended in fresh sterile saline solution to reach an initial concentration of 1.5 × 10^8^ CFU/mL (equivalent to 0.5 McFarland standard).

One hundred microliters of the 1:100 diluted cell suspensions (to reach a concentration of 1.5 × 10^6^ CFU/mL) were dispensed into each well of a 96-well microtiter plate containing 100 μL of Mueller Hinton broth (Thermo Fisher Diagnostics S.p.A, Rodano, Italy). The strains were exposed to 2-fold dilution series of each product. After incubation for 24 h at 37 °C, the MICs were read as the lowest dilution of molecules able to inhibit visible bacterial growth. For all strains, MIC was evaluated against Ciprofloxacin (range 64–0.03 mg/mL) (Merk Life Science S. r. l, Milan, Italy) as a positive antimicrobial efficacy control. All the experiments, for each molecules and each microorganism, were performed in duplicate.

### Fungal Strains and Culture Conditions

The assays have been performed on 10 fungal strains, which belong to 7 different species: *Pyricularia oryzae* – A252 and PO21_07; *P. expansum* EM22, *A. niger* ITEM7096 and ITEM7097, *M. pyriformis* EM14, *B. cinerea* EM1, *F. culmorum* FcUK and *F. graminearum* ITA1601. They have been identified by ITS sequencing and stored in Malt Agar medium (MA) (20 g/L of malt extract (Oxoid, Hempshire, UK), 15 g/L agar (Applichem, Darmstadt, Germany) and Milli-Q water). All the media have been sterilized in an autoclave.

Fungi have been classified according to their growth rate into fast-growing (*M. pyriformis* EM14, *B. cinerea* EM1, *F. culmorum* FcUK and *F. graminearum* ITA1601) and medium-speed growers (*P. oryzae* A252 and PO21_07; *P. expansum* EM22, *A. niger* ITEM7096 and ITEM7097).

All strains have been renewed on MA medium before the assay on Petri dishes. Small pieces of fungal colonies were transferred to a new MA medium in which the tested compounds had been added at 25 mg/L concentration. All tested compounds have been solubilized in DMSO before the addition to the medium. Azoxystrobin (Amistar, Syngenta) was used as a reference compound of fungal growth inhibition at a concentration 25 mg/L a.i.

MA medium was used as a control for azoxystrobin and MA + 1% DMSO for the tested compounds. For each strain, two replicas have been prepared and the fungi were incubated at 24 °C. The growth of the colonies was measured on days two and four postinoculation for the fast-growing fungi and on days 6 and 7 for the medium-growing ones. The inhibition was calculated by comparing the growth on the control medium and the compound-added medium.

## Results and Discussion

### CaL-B-Mediated Preparation of Benzoyl and Cinnamoylamides

Although there are many methodologies to prepare amides, the main focus of current research regards the development of green biocatalytic approaches for the synthesis of natural phenylamides reducing waste and overall costs.^24,25^ Lipases [EC 3.1.1.3] are versatile biocatalysts which present excellent catalytic characteristics such as high selectivity, high stability, lack of cofactor dependency, while requiring mild reaction conditions.^26−28^ Among them, the lipase B from *Candida antarctica* presents extreme properties (*e.g.*, organic solvent and high temperature tolerance) as well as a broad spectrum of applications compared to other lipases. The 3D structure of CaL-B was first elucidated by Uppenberg et al.^29,30^ revealing that the active site consists of amino acids Asp 187, His 224, Ser 105 which are the typical catalytic triad of hydrolases. Its mechanism is referred to as “bi-bi ping-pong” and consists of a serine reaction with carboxylic acids or their derivatives (*e.g.*, esters) and production of an acyl-enzyme intermediate. Activated by the acyl-enzyme formation and the hydrogen bonds of nearby amino acidic residues, a nucleophilic attack occurs, and the product is finally released, regenerating the enzyme which is ready for another reaction cycle.

Following the paper of Aftab and co-workers,^31^ a first set of amides has been prepared via a CaL-B mediated biotransformation using commercially available ethyl esters (**1a**–**c**) and tryptamine (Figure 2). High substrate loading (0.25 M for the acyl donor and 0.5 M for the amines), and imm-CaL-B (50 mg/mL) were employed, obtaining the desired products **2a**–**c** in modest-to-good yields (15–77%). No reaction was detected using the trisubstituted ethyl 3,4,5-trimethoxybenzoate as acyl donor, possibly due to its steric hindrance in the catalytic pocket. To avoid any water interference, molecular sieves were added to the reaction mixture. Considering the optimal solubility of the starting materials together with the remarkable enzyme tolerance to high temperature and organic solvents, the reactions were performed at 80 °C in *tert*-amyl alcohol (*t*-AA) as reaction medium. *t*-AA is considered a green solvent, as it derives from fusel oil, which is a byproduct of the ethanol production from sugars.^32^ Furthermore, by simple standard operations (i.e., filtration and solvent removal) both the catalyst and *t*-AA can be recovered and reused for several cycles, further enhancing the sustainability of the proposed process. It is worth noting that classical chemical protocols for amide preparation generally require the use of unstable coupling agents, harsh reaction conditions, as well as high energy consumption. In this context, our biocatalytic approach can be considered a sustainable alternative to traditional chemical synthesis, especially if combined with unconventional green media, catalyst recycling, and solvent recovery-and-reuse strategies.

**Figure 3:**
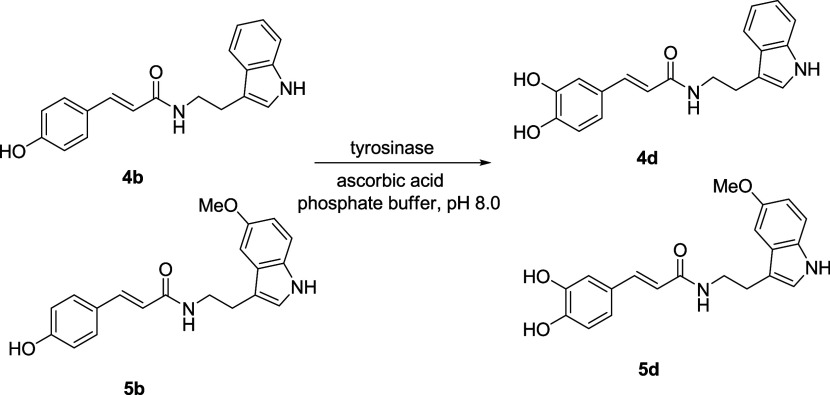
Tyrosinase-mediated preparation of 4d and 5d.

Mimicking natural plant pathways, the enzymatic preparation of cinnamoyltryptamines was subsequently carried out (Figure 2). Compounds **4a**–**c** and **5a**–**c** were produced in modest-to-very-good yields (20–80%), using the reaction conditions previously described. Since no reaction was observed by employing etlyl caffeolate as CaL-B acyl donor following our previous optimized reaction conditions, a tyrosinase-mediated biotransformation was set up to obtain **4d** and **5d** (Figure 3).^33,34^ More in detail, 10 mM of **4b** or **5b** were biotransformed exploiting a selective oxidation mediated by tyrosinase to give catechols **4d** and **5d**. To avoid overoxidation, ascorbic acid was added to the reaction medium (3 equiv).

The potential antimicrobial activity of the obtained benzoyl (**2a**–**c**) and cinnamoylamides (**4a**–**d**, **5a**–**d**,) were subsequently assessed (Tables 1 and 2).

**Table 1:** Antifungal Activity of Benzoyl (**2a**–**c**) and Cinnamoylamides (**4a**–**d**, **5a**–**d**)^a^

**molecule**	**Po wt**^b^**inhibition (%)**	**Po res**^b^**inhibition (%)**	**Pe**^b^**inhibition (%)**	**An96**^b^**inhibition (%)**	**An97**^b^**inhibition (%)**	**Mp**^c^**inhibition (%)**	**Bc**^c^**inhibition (%)**	**Fc**^c^**inhibition (%)**	**Fg**^c^**inhibition (%)**
**2a**	1.5	31.3	23.5	9.7	10.8	–3.7	9.6	–9.7	–2.1
**2b**	26.9	35.1	10.6	4.0	10.0	23.2	29.6	12.3	–34.0
**2c**	6.0	13.0	5.3	–0.8	–1.5	34.2	3.3	–4.5	–21.3
**4a**	21.1	26.9	14.0	14	0.7	12.5	28.1	11.3	5.2
**4b**	23.9	30.2	14.7	3.4	4.6	15.4	32.8	13.6	4.3
**4c**	1.3	7.3	5.7	3.8	5.5	12.0	18.6	4.6	–1.0
**4d**	0.7	3.3	4.3	4.4	3.7	5.3	3.0	4.2	–1.4
**5a**	12.7	20.1	7.7	2.7	2.6	–11.0	17.8	5.0	1.4
**5b**	15.5	20.1	8.4	4.7	4.0	–9.6	20.2	11.2	3.8
**5c**	0.7	6.7	5.7	1.9	1.8	8.7	4.2	1.7	–1.4
**5d**	1.3	4.7	9.3	3.8	6.1	5.3	6.8	5.8	4.8
**azoxystrobin**	96.5	52.5	68.7	55.3	47.1	40	72.4	54.9	51.7

a *Po* wt: *P. oryzae* A252; *Po* res: *P. oryzae* PO21_07; *Pe*: *P. expansum* EM22; *An* 96: *A. niger* ITEM7096; *An* 97: *A. niger* ITEM7097; *Mp*: *M. pyriformis* EM14; *Bc*: *B. cinerea* EM1; *Fc*: *F. culmorum* UK; *Fg*: *F. graminearum* ITA1601.

b After 7 days of growth (medium-speed growing fungi).

c After 4 days of growth (fast-growing fungi).

**Table 2:** Antibacterial Activity of Benzoyl (**2a**–**c**) and Cinnamoylamides (**4a**–**d**, **5a**–**d**)^a^

	***Ec***	***Se***	***Pa***	***Sa***
**molecule**	**MIC (μM)**	**MIC (μM)**	**MIC (μM)**	**MIC (μM)**
**2a**	385.2	385.2	385.2	192.6
**2b**	459.8	459.8	459.83	229.92
**2c**	394.6	394.6	394.6	197.3
**4a**	881.6	440.8	440.8	440.8
**4b**	835.5	835.5	835.5	835.5
**4c**	380.5	380.5	380.5	380.5
**4d**	397.0	794.0	794.0	794.0
**5a**	799	399.5	399.5	399.5
**5b**	761.0	380.5	761.0	761.0
**5c**	349.3	349.3	349.3	349.3
**5d**	363.6	363.6	363.6	90.9
**ciprofloxacin**	0.1	0.1	1.5	1.5

a *Ec* = *E. coli*; *Se* = *S. enterica* Enteritidis; *Pa* = *P. aeruginosa*; *Sa* = *S. aureus*.

In recent literature reports, a series of natural benzoic and cinnamic acids were evaluated against a variety of different fungi and bacteria, presenting MICs in the range of 0.3–8 mM.^35^ Therefore, the newly generated benzoyl and cinnamoylamides were screened against a panel of medium- and fast-growing fungal plant pathogens as well as Gram-positive and Gram-negative foodborne bacteria. The results are expressed as inhibition of growth (%) in Table 1, while they are presented as MIC against target microorganisms in Table 2. Interestingly, benzoyltryptamines **2a**–**c**, Table 1, show more than 30% growth inhibition on a resistant strain of *P. oryzae* as well as *M. pyriformis*. Cinnamoyl derivatives **4a** and **4b** present antifungal properties (≈30% growth inhibition) against the resistant strain of *P. oryzae* and wild-type *B. cinerea*, while **5d** shows an appreciable activity against *S. aureus* (MIC: 90.9 μM). According to the obtained results, minimal structural modifications lead to different and not easily predictable bioactivity variations. In light of these considerations, these molecules can be considered as a starting point for further development of natural phytoalexin-inspired antimicrobial agents.

In summary, a convenient biocatalytic approach was developed for the preparation of a series of benzoyl and cinnamoylamides which have been tested for their antimicrobial properties against a panel of phytopathogenic fungi and foodborne bacteria. Among benzoylamides, **2a**–**c** were active against a resistant strain of *P. oryzae* as well as against *M. pyriformis*, while the cinnamoylamides **4a** and **4b**, demonstrated antifungal properties on a resistant *P. oryzae* and wild-type *B. cinerea*. **5d**, characterized by a cathecol moiety and 5-methoxytryptamine, showed antimicrobial activity against the Gram-positive bacterium *S. aureus.* The synthesized compounds represent promising natural phytoalexin-inspired prototypes, which could serve as a basis for further research in the field of antimicrobials. The use of CaL-B in the green solvent *t-*AA allowed us to overcome any solubilization issue of the starting materials, giving rise to productive strategies (15–80% isolated yield) and demonstrating high stability of the enzyme in organic solvents and high temperature (80 °C).

## Supporting Information Available

^1^H NMR and ^13^ C NMR of the generated compounds (Figures S1–S11) (PDF)
