# Supplementary material for: Biocatalyzed Synthesis of Benzoyl and Cinnamoylamides Inspired by Rice Phytoalexins
Source: ACS Agric Sci Technol. 2025 Feb 24;5(4):461–7. doi: 10.1021/acsagscitech.4c00380 (PMC12015818; doi:10.1021/acsagscitech.4c00380)
Supplement: Supplementary file 1 — as4c00380_si_001.pdf [file as4c00380_si_001.pdf]

Supporting information

Biocatalyzed synthesis of benzoyl and cinnamoylamides inspired by rice phytoalexins

Cecilia Pinna,[a‡] Luca Nespoli,[a‡] Giulia Brioschi,[a] Andrea Kunova,[a] Paolo Cortesi,[a] Piera Anna Martino,[b] Francesco Molinari,[a] Loana Musso,[a] Sabrina Dallavalle,[a] Martina L. Contente,[a*] Andrea Pinto[a]

[*] corresponding author e-mail: martina.contente@unimi.it

‡ These authors contributed equally to this work

[a] *Department of Food, Environmental and Nutritional Sciences (DeFENS), University of Milan, via Celoria 2, 20133 Milan, Italy*

[b] *Department of Biomedical, Surgical and Dental Sciences (DSBCO), One Health Unit, University of Milan, via Pascal 36, 20133 Milan, Italy*

Table of contents

1. NMR spectra 2

1. NMR spectra

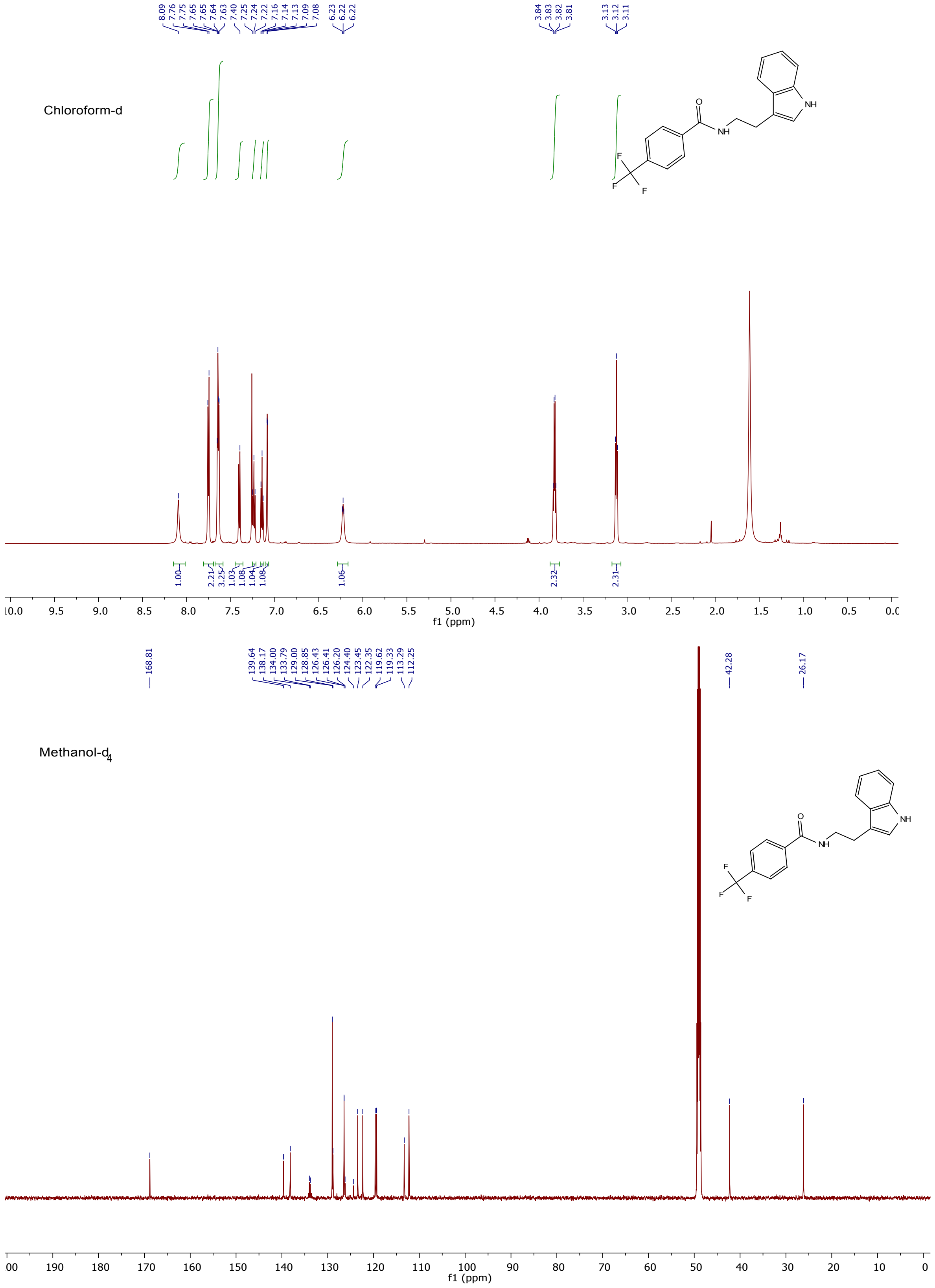

Figure S 1. ^{1}H-NMR ^{13}C-NMR of compound **2a**

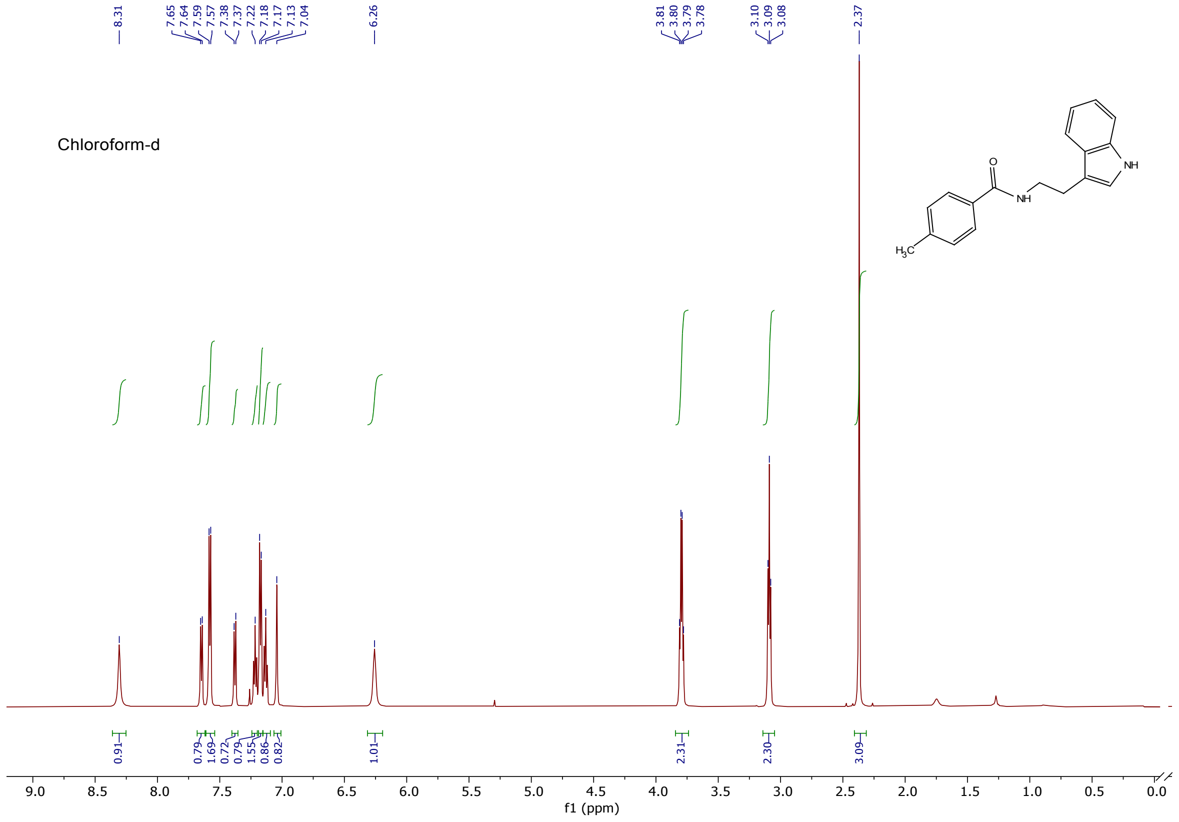

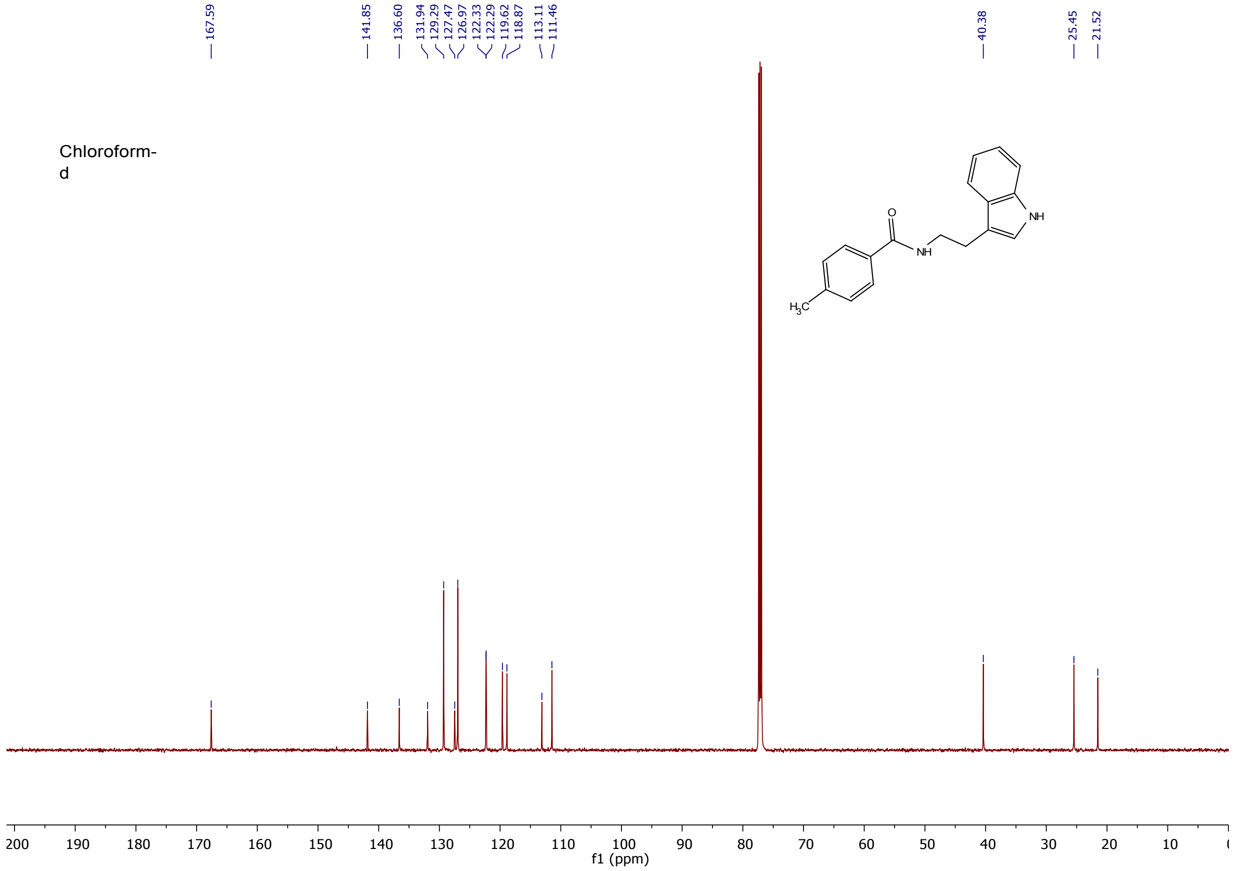

Figure S 2. ^{1}H-NMR ^{13}C-NMR of compououund **2b**

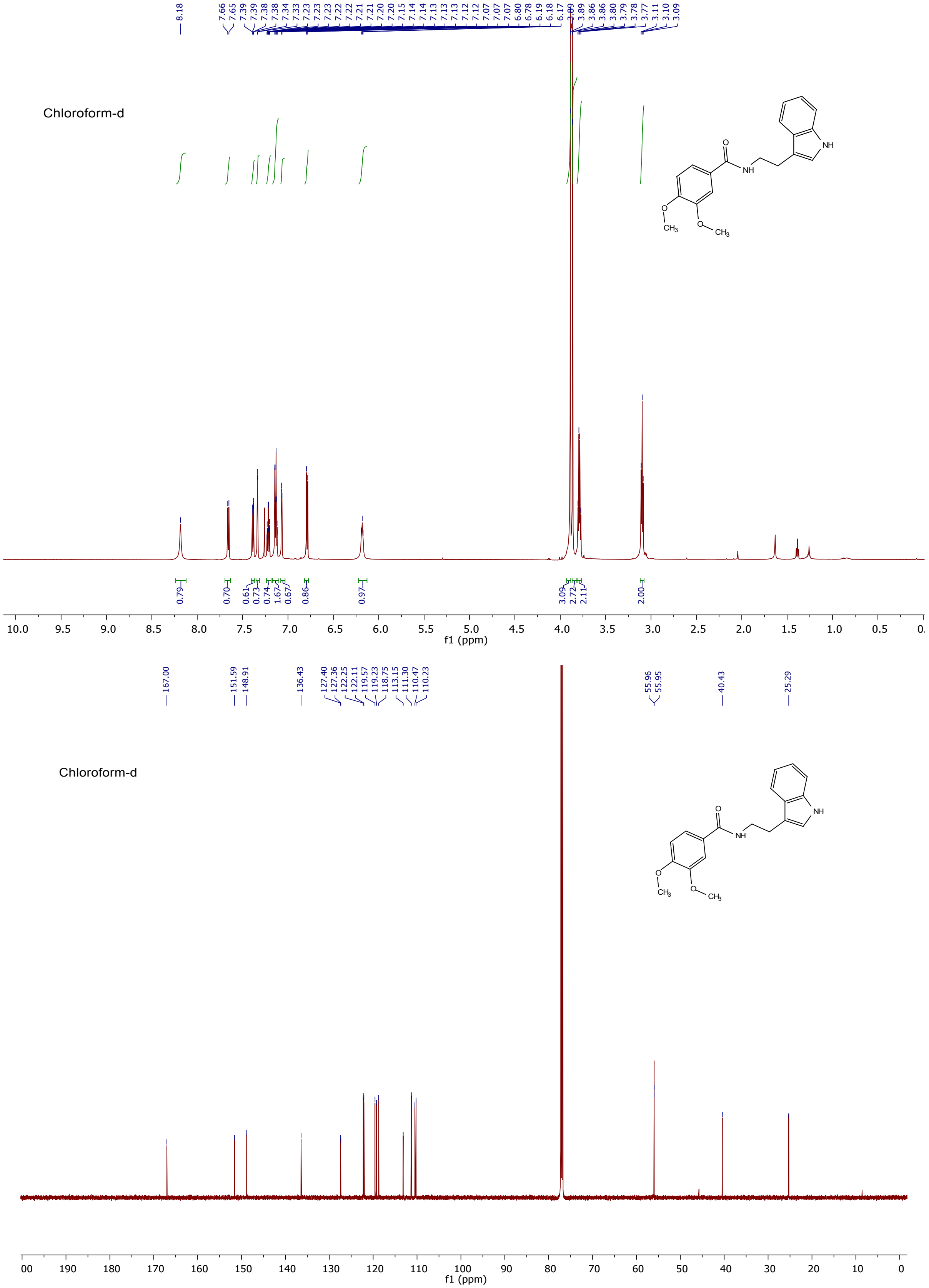

Figure S 3. ^{1}H-NMR ^{13}C-NMR of compound **2c**

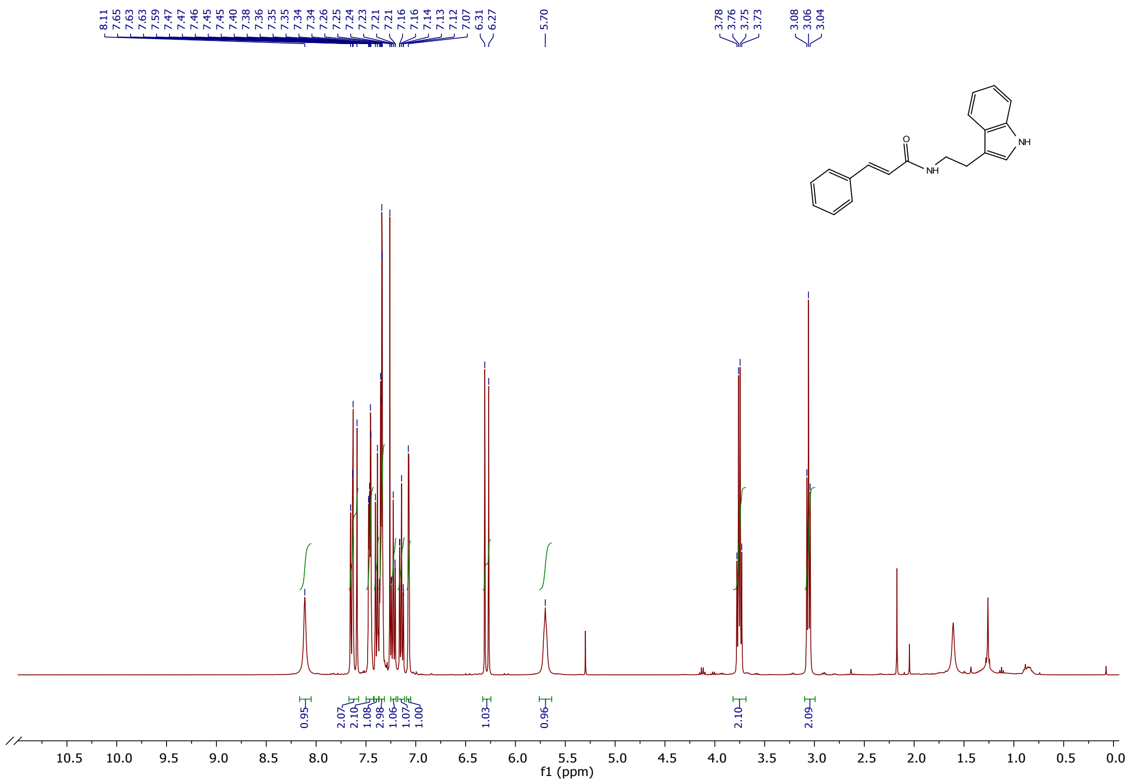

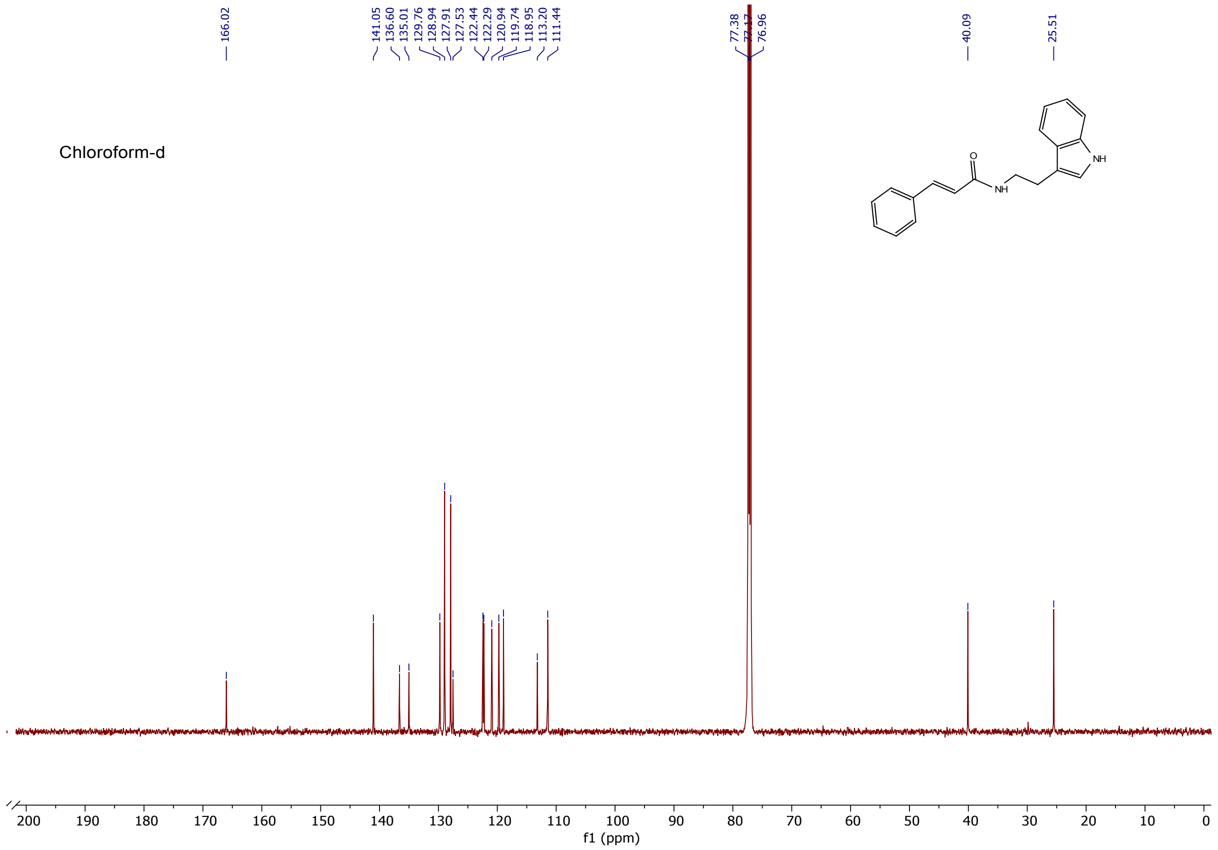

Figure S4. ^{1}H-NMR ^{13}C-NMR of compound **4a**

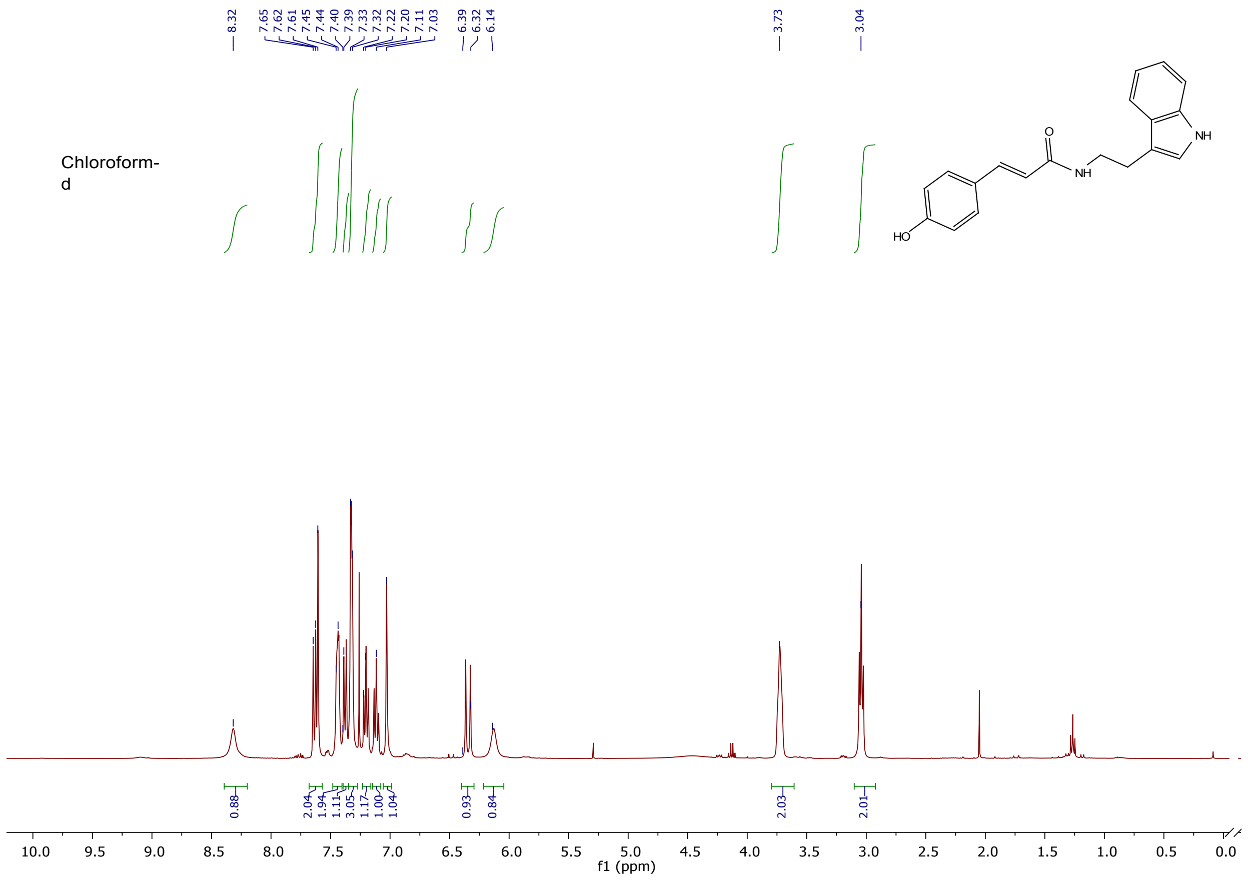

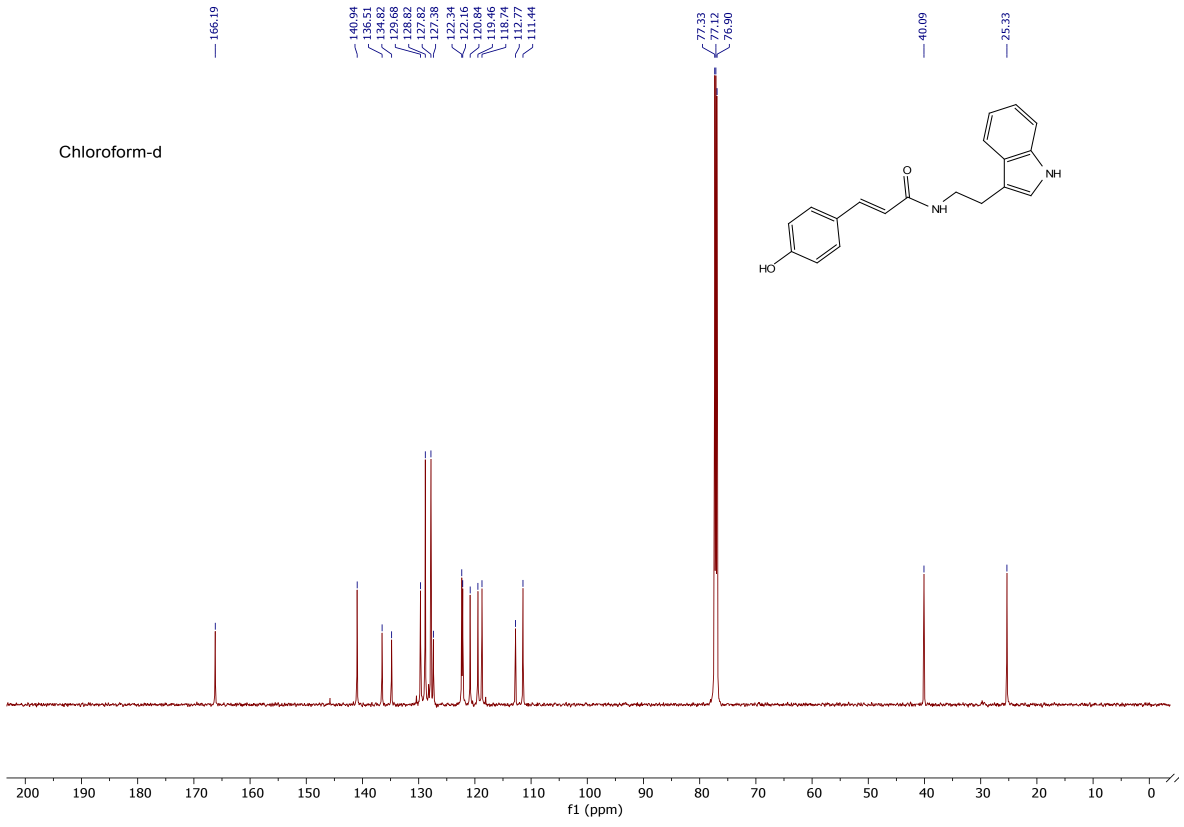

Figure S 5. ^{1}H-NMR ^{13}C-NMR of compound **4b**

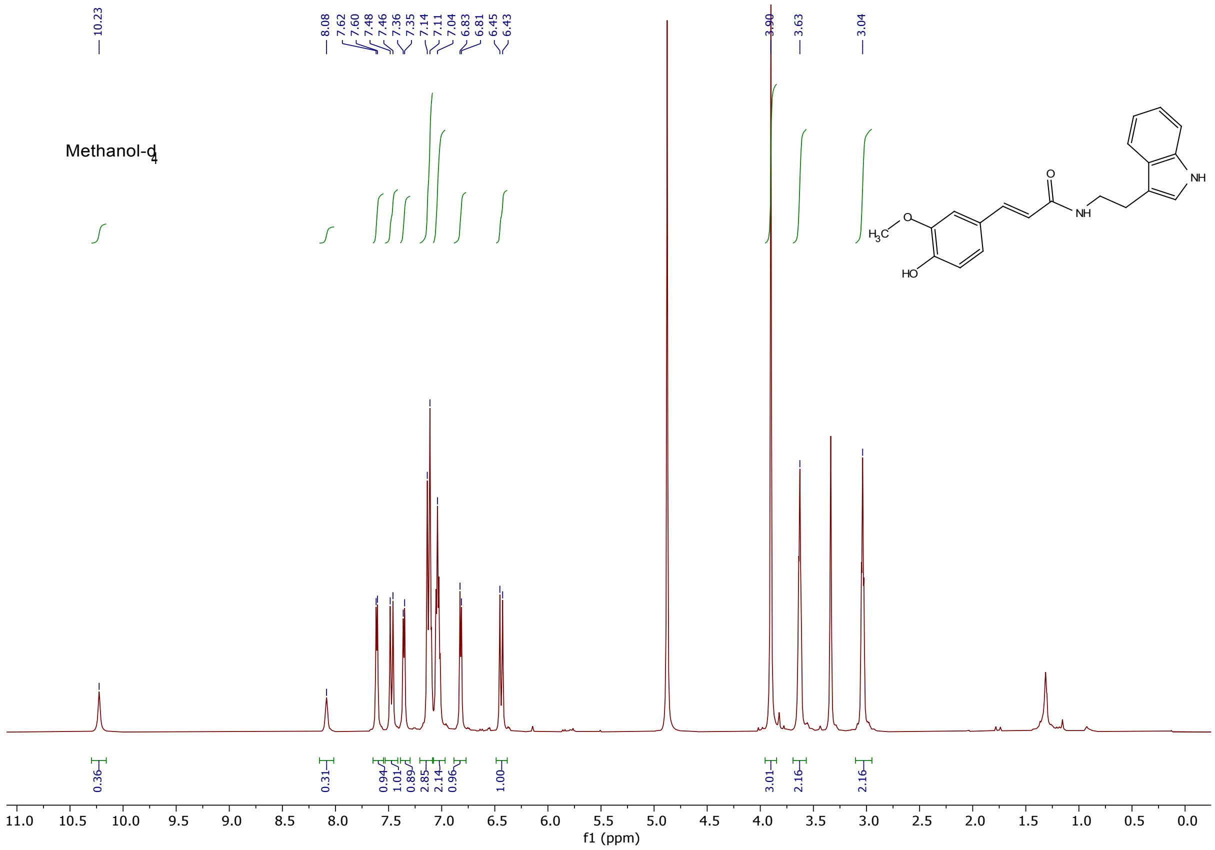

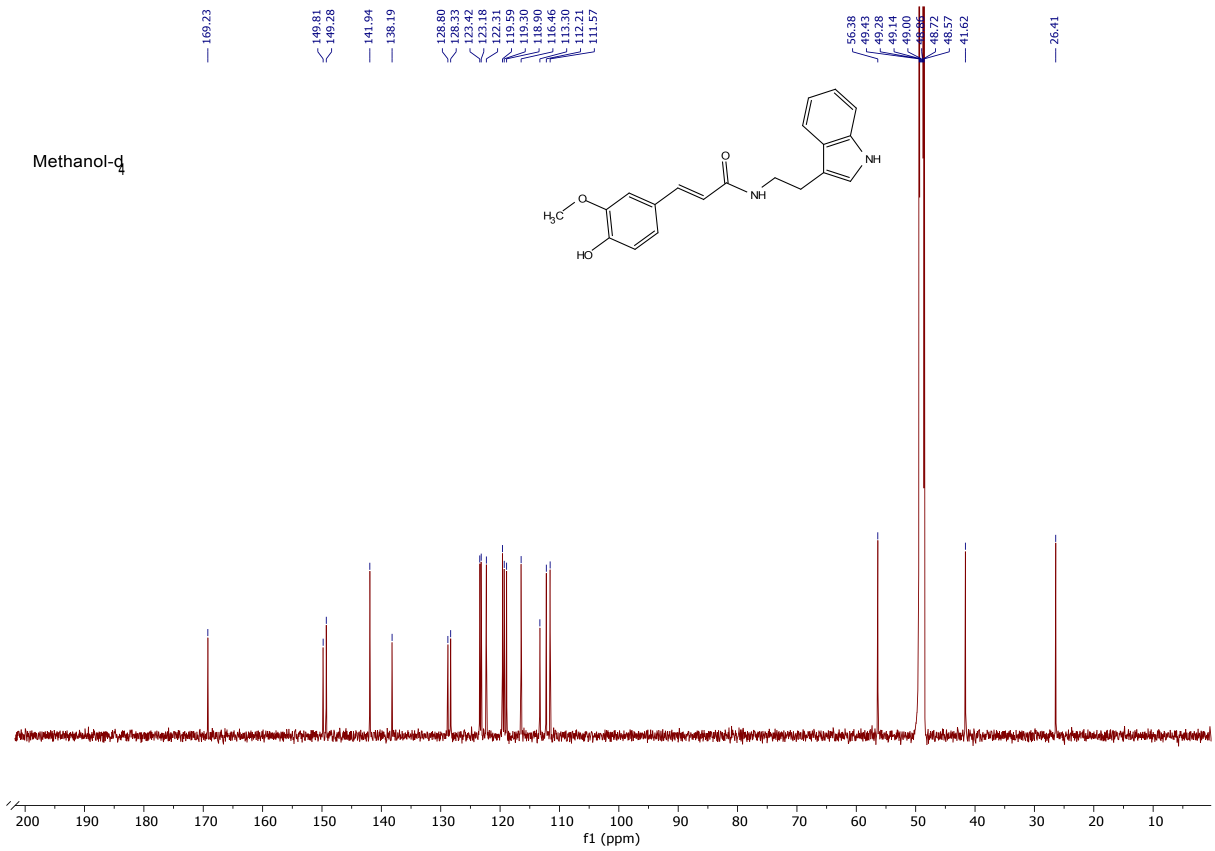

Figure S 6. ^{1}H-NMR ^{13}C-NMR of compound **4c**

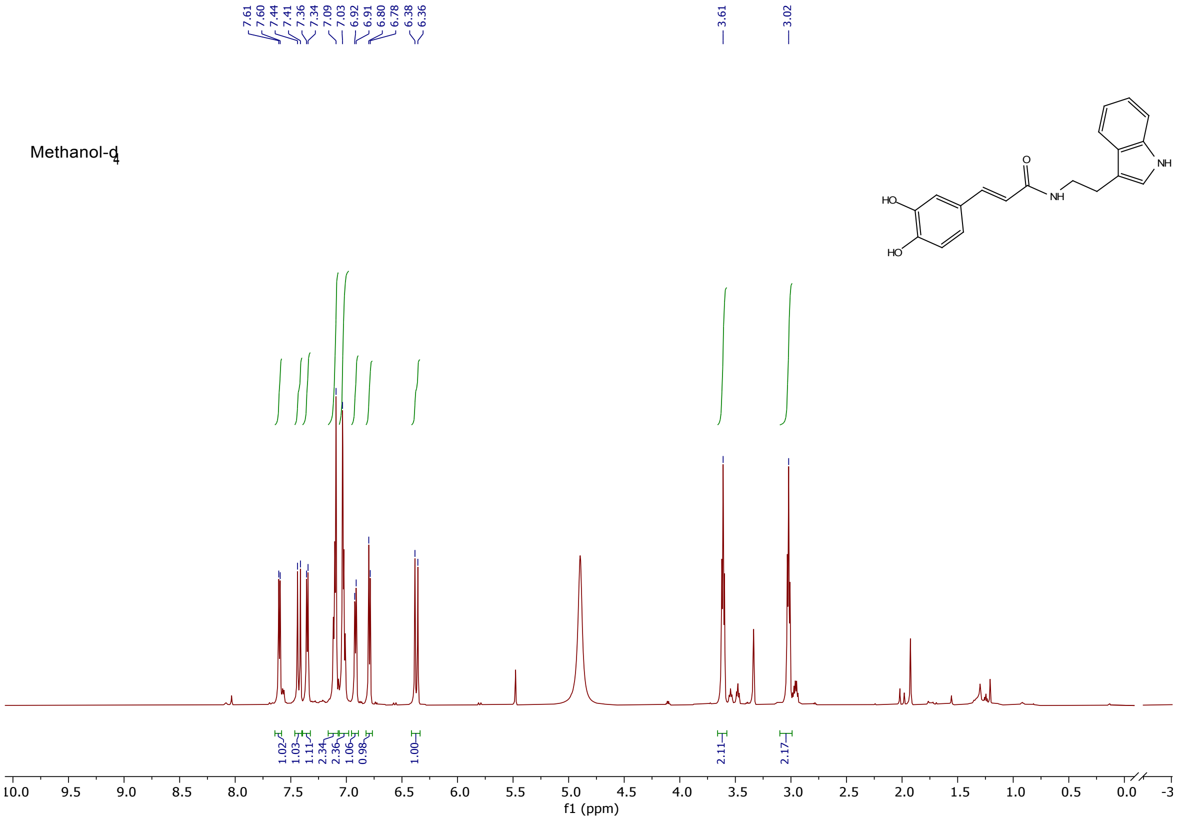

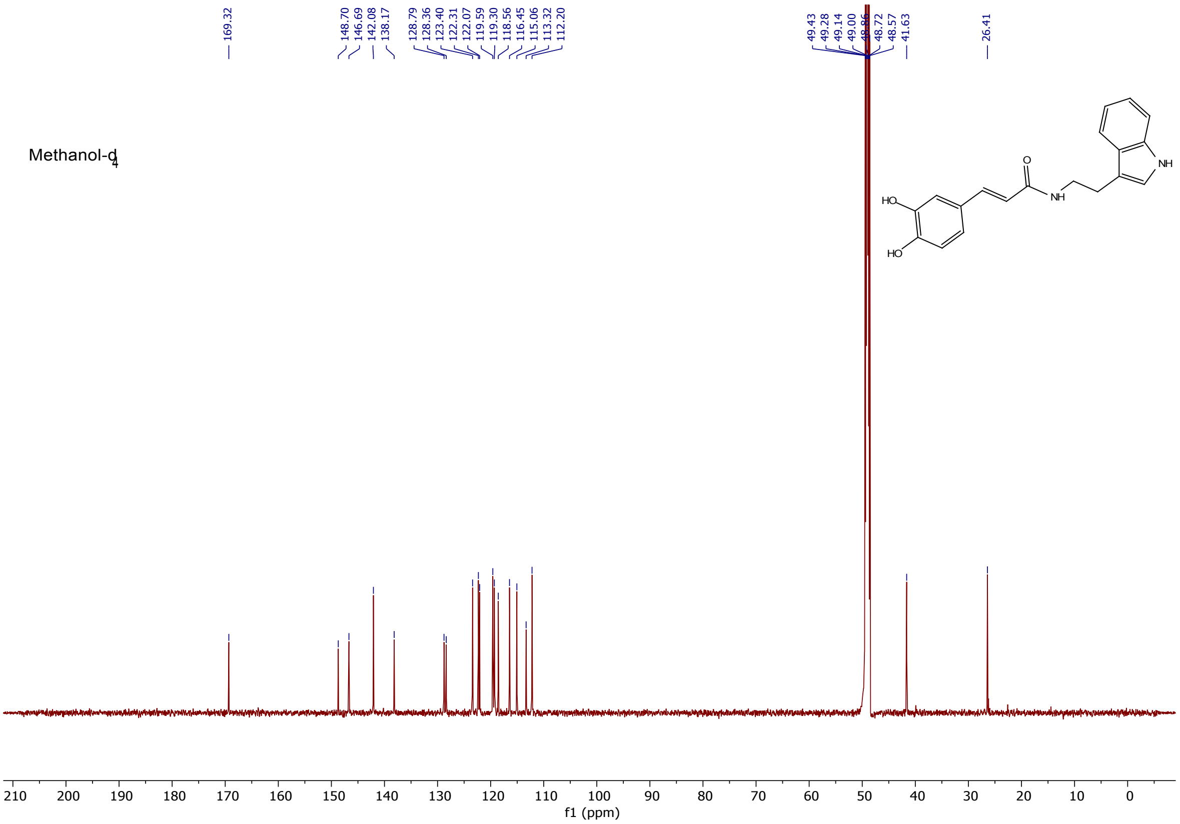

Figure S 7. ^{1}H-NMR ^{13}C-NMR of compound **4d**

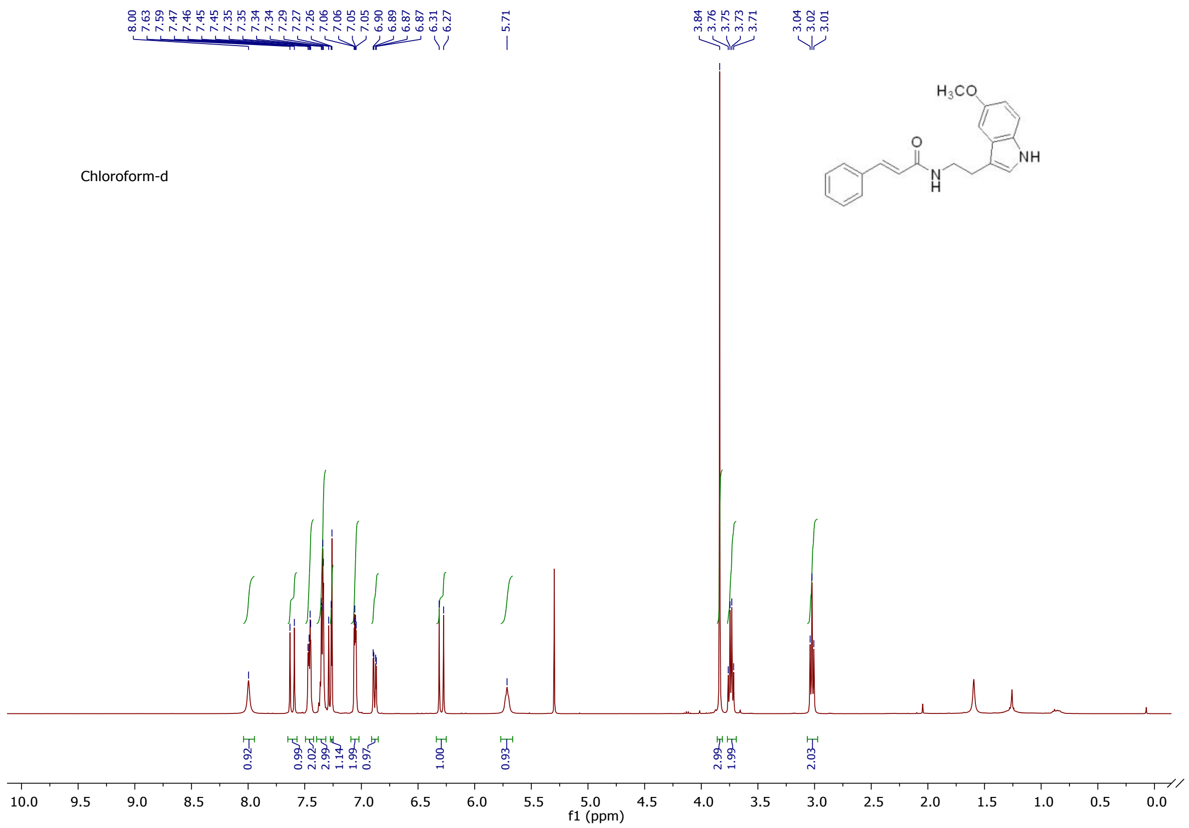

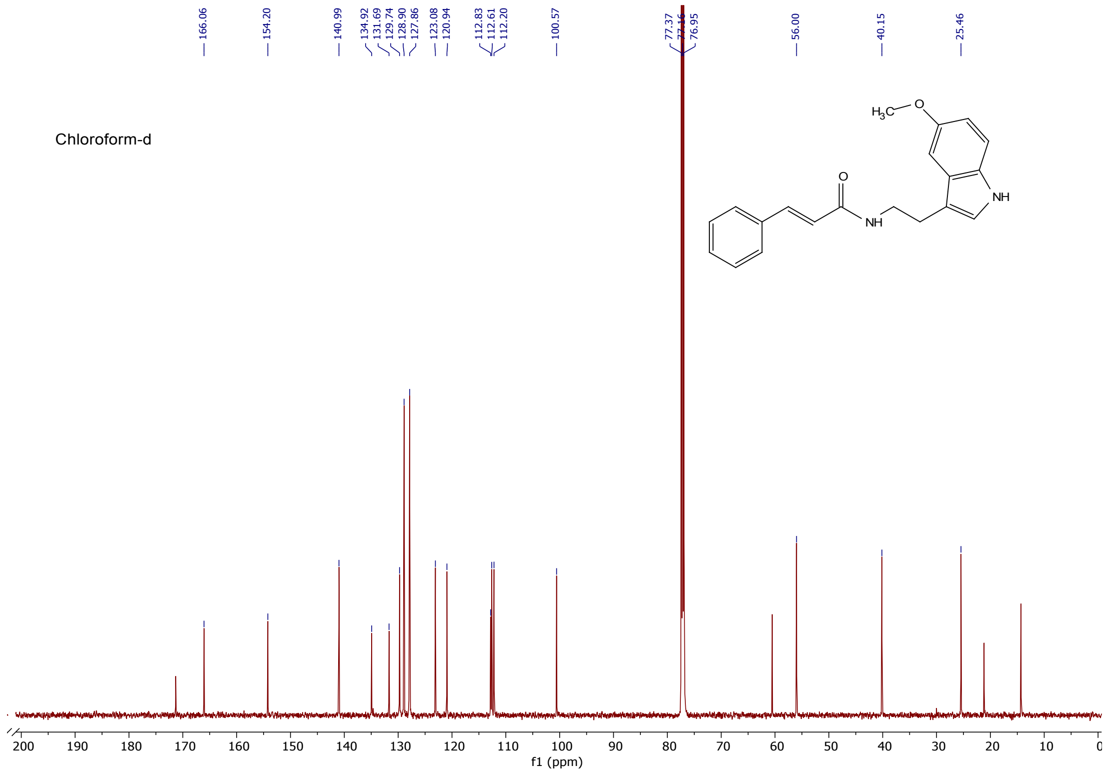

Figure S 8. ^{1}H-NMR ^{13}C-NMR of compound **5a**

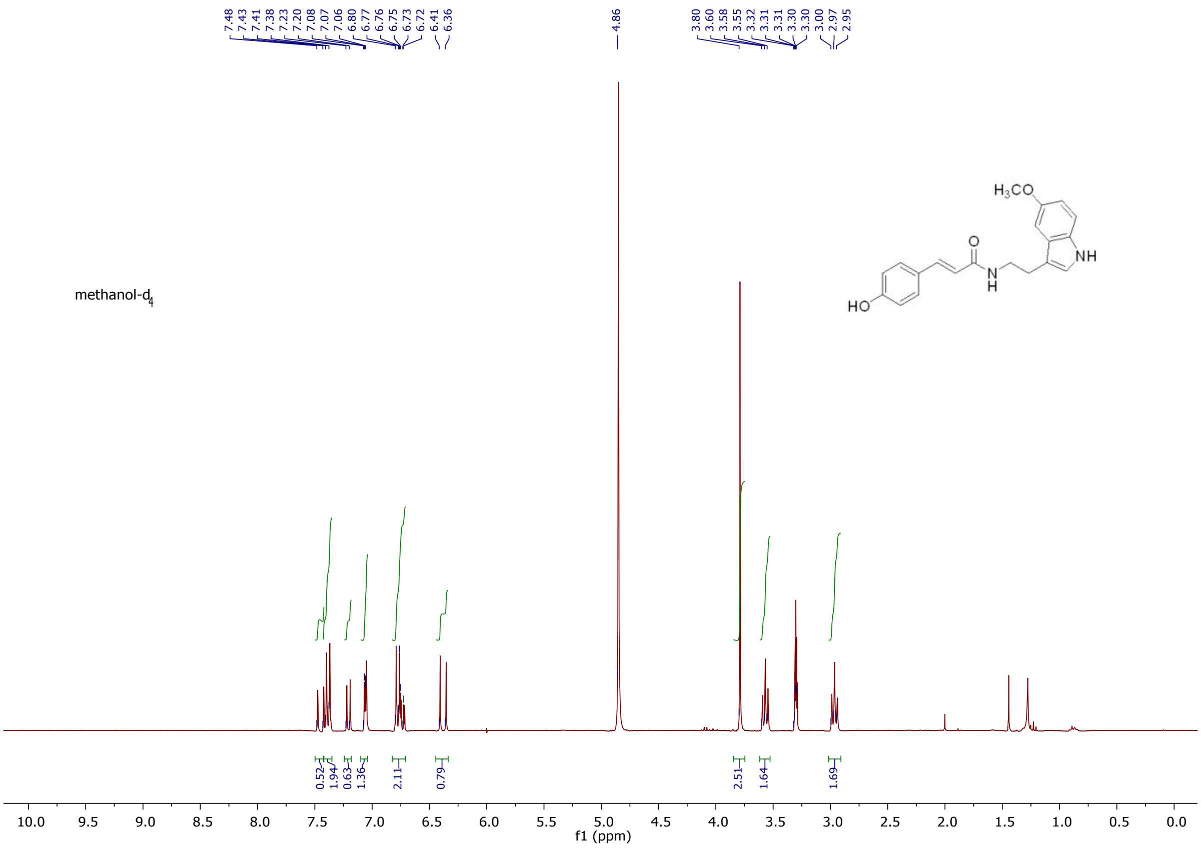

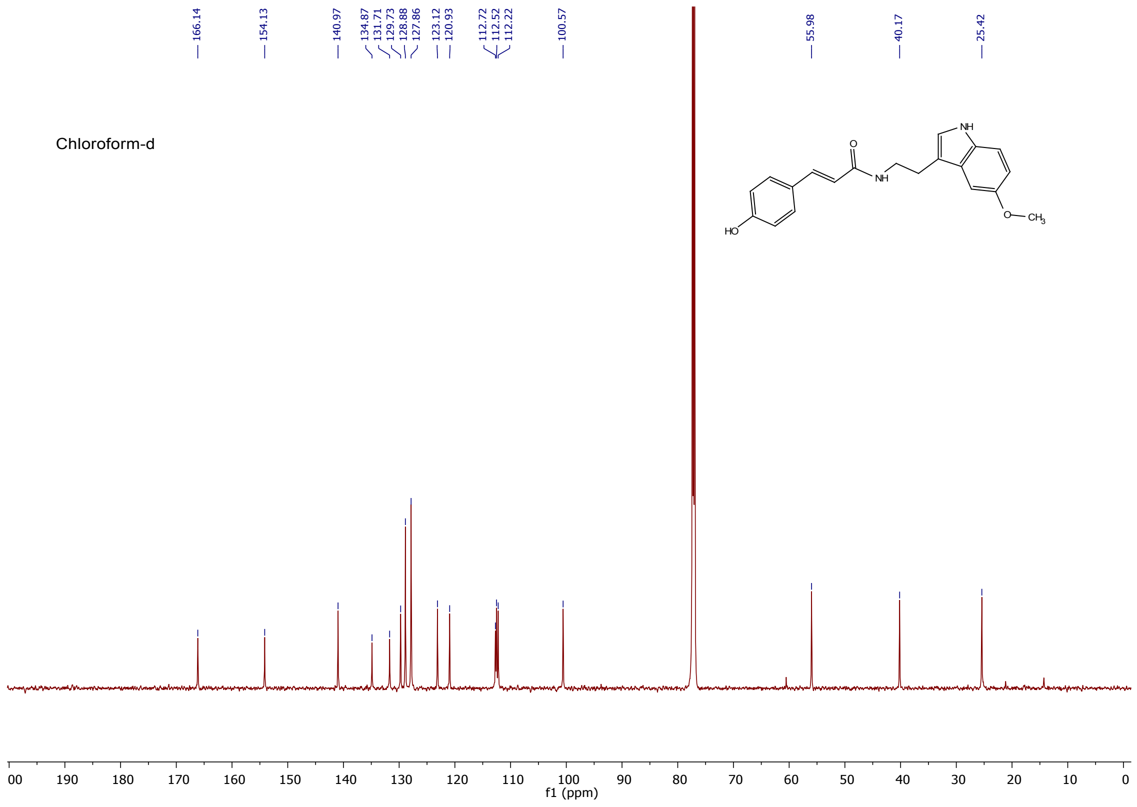

Figure S 9. ^{1}H-NMR ^{13}C-NMR of compound **5b**

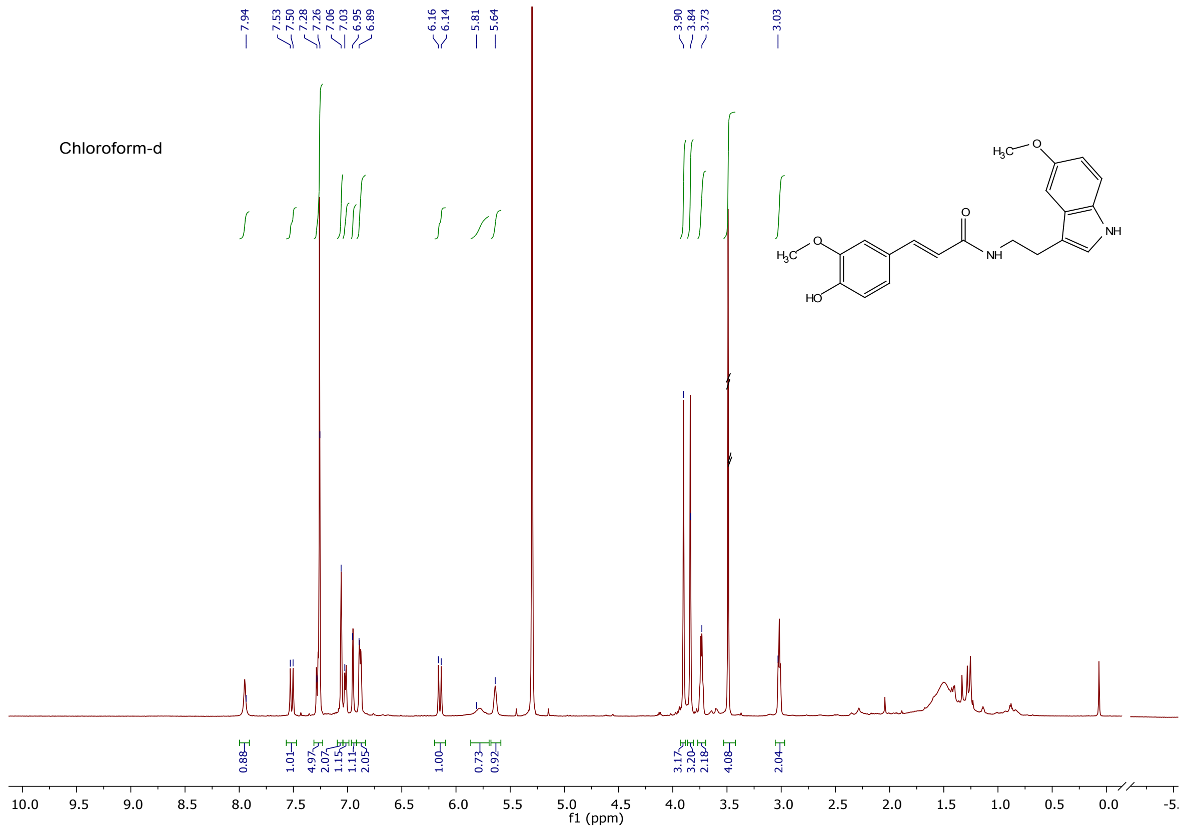

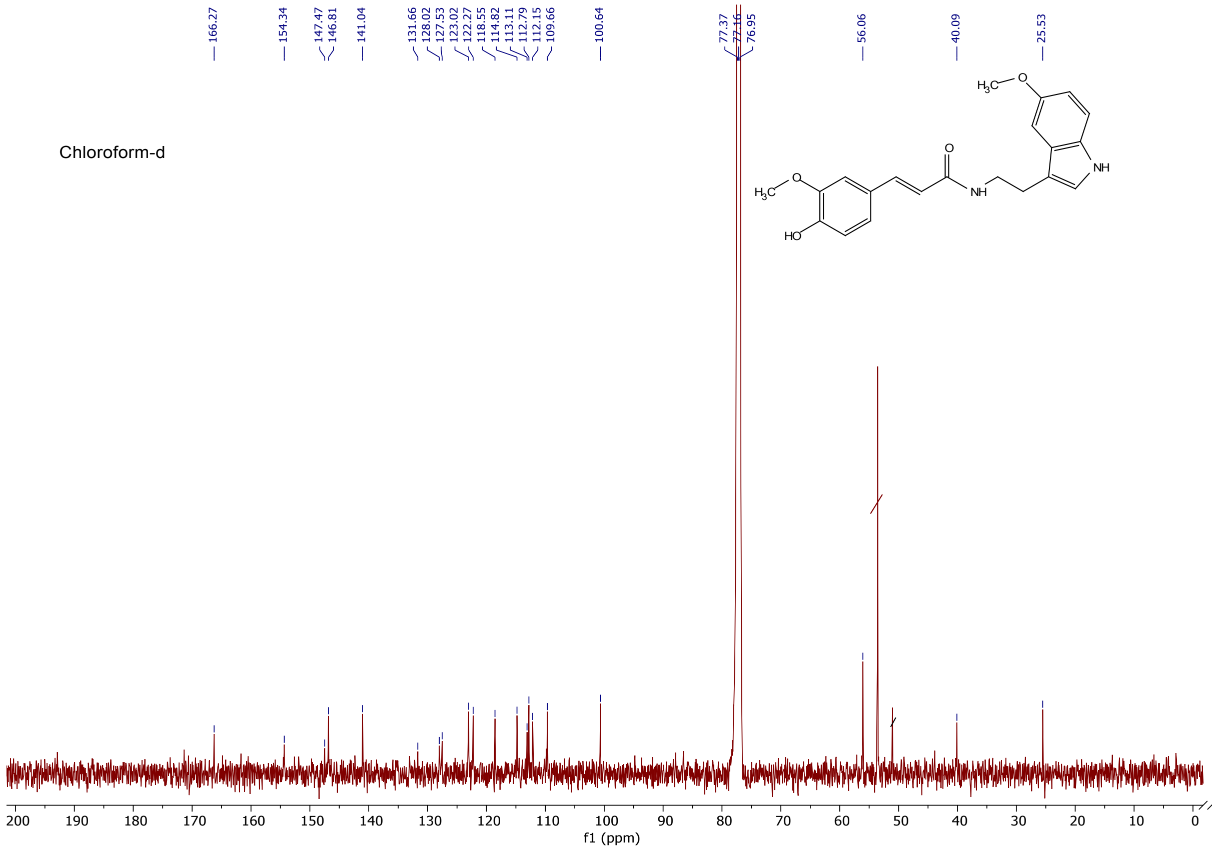

Figure S 10. 1H-NMR 13C-NMR of compound **5c**

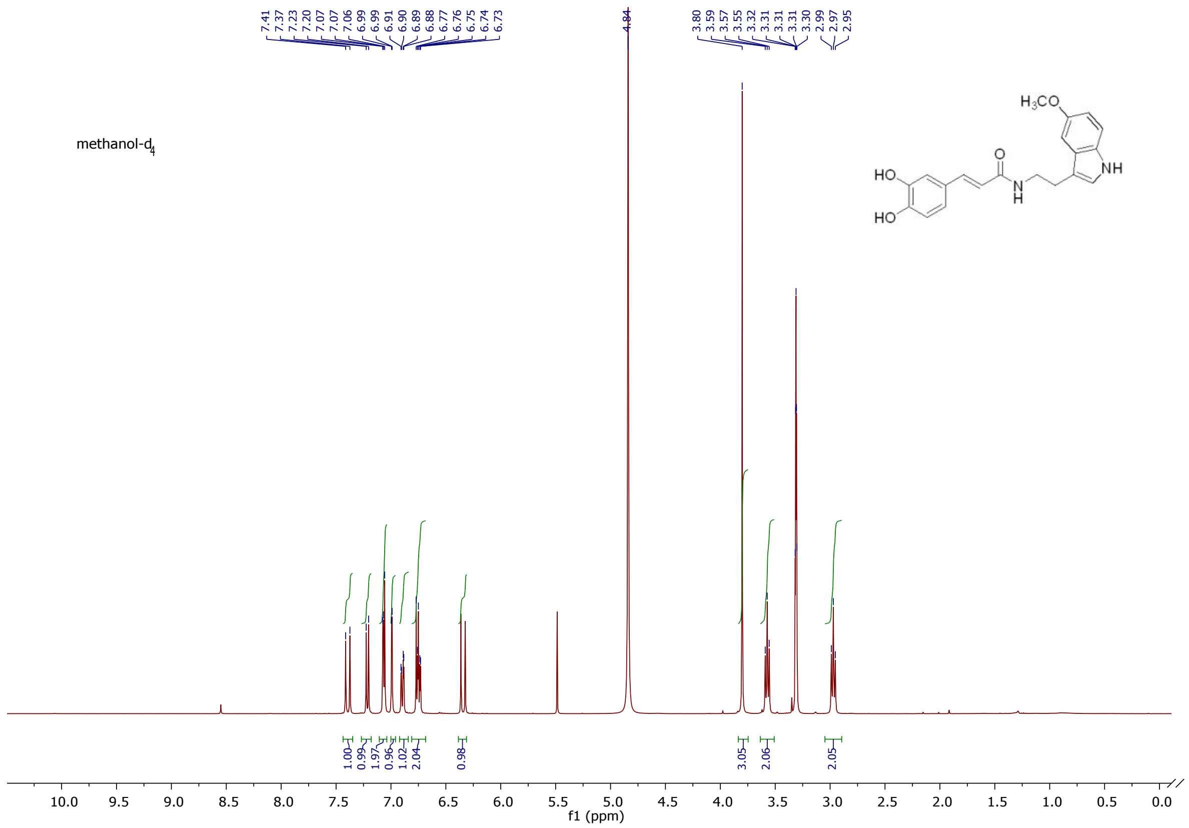

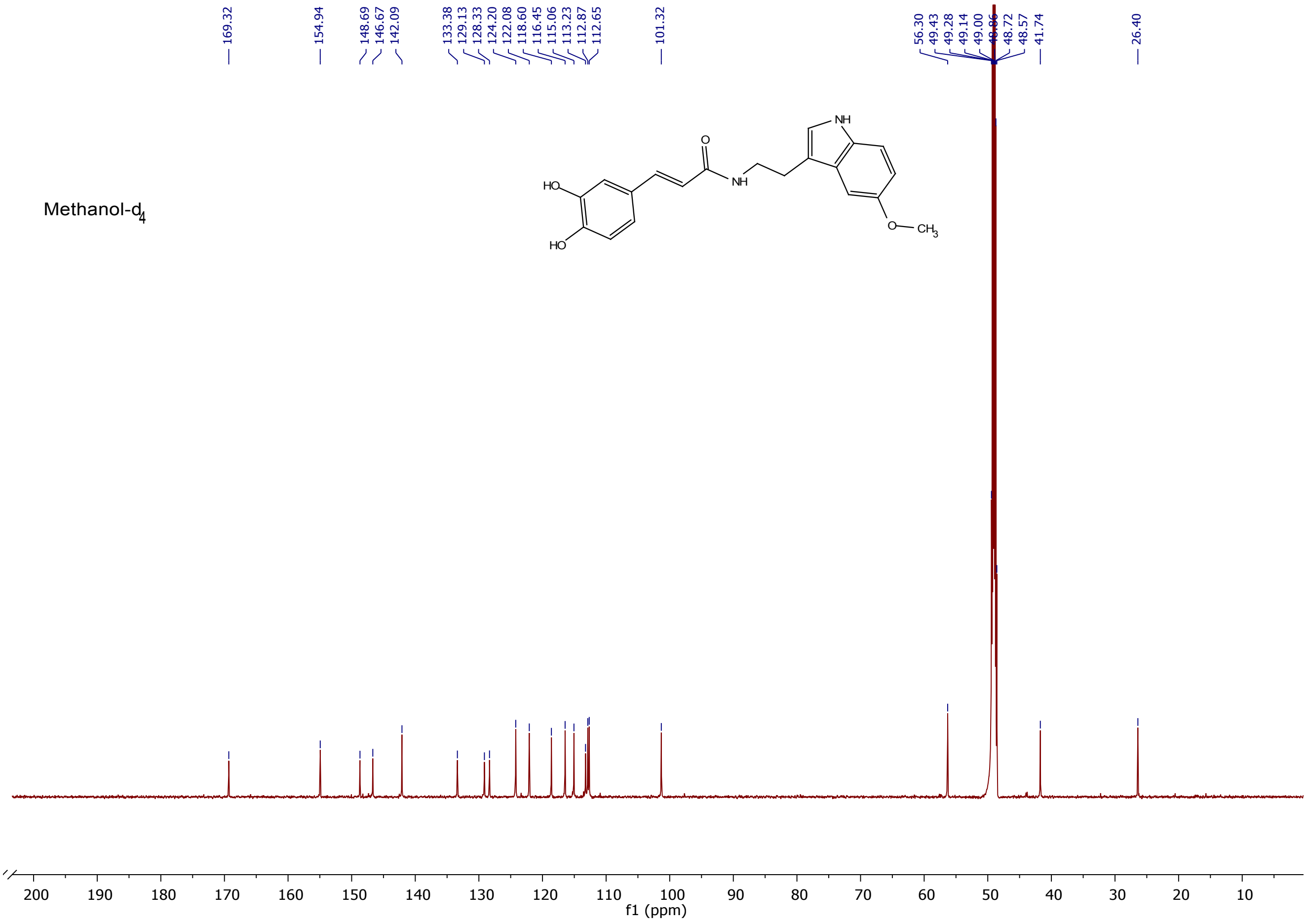

Figure S 11. ^{1}H-NMR ^{13}C-NMR of compound **5d**
